# High Precision Timing with Parabolic Equation Fitting in Narrowband Systems †

**DOI:** 10.3390/s19194164

**Published:** 2019-09-25

**Authors:** Jun Zou, Chen Xu

**Affiliations:** School of Electronic and Optical Engineering, Nanjing University of Science and Technology, Nanjing 210094, China; chen_xu@njust.edu.cn

**Keywords:** timing detection, narrowband system, parabolic equation fitting

## Abstract

Timing forms the basis of wireless communication systems. Orthogonal frequency division multiplexing (OFDM) technology has strict requirements for synchronization performance, and timing errors lead to interference between subcarriers and symbols. Although cyclic prefix (CP) can relax the timing requirement, high precision timing is still necessary and can release the pressure on CP. Due to the uncertainty of signal arrival, there is a sampling offset between the sampling sample’s timing and the real timing, which can be large in the narrowband system with a low sampling rate. In this paper, we propose a parabolic equation fitting method to improve the timing precision in narrowband systems that have two times the rate of the Nyquist sampling rate. The proposed timing method is easy to implement, with low additional complexity compared to traditional timing detection and is based on traditional direct correlator output.

## 1. Introduction

The Internet of Things (IoT), which aims to connect all the physical objects in the world, has gained a lot of attention in recent years [1]. Wireless cellular communication, which can perfectly support machine to machine communication and has a large coverage, forms the basis of the IoT. According to the characteristics of the IoT application scenario, it can be divided into massive IoT and mission-critical IoT [2,3,4]. In mission-critical IoT, low latency and high transmission data rates are the most important requirements, while in massive IoT, the number of access devices can be very large so low cost is necessary. Moreover, the packet size is usually small, and its transmission frequency is low for the majority of massive IoT applications such as smart meters and remote sensors [5,6]. Therefore, narrowband transmission is a good choice for massive IoT as it can reduce the radio frequency (RF) cost and achieve large coverage with high spectrum efficiency.

A lot of research has been done on narrowband communication technologies [7,8,9,10,11]. There are a lot of low power wide area network (LPWAN) technologies with narrowband transmission on unlicensed spectrums that have been proposed for massive IoT applications in recent years, such as LoRa and Sigfox [12]. The 3rd Generation Partnership Project (3GPP) also establishes the narrowband-IoT (NB-IoT) standard on the licensed spectrum from release 14 [13]. LPWAN has become one of the main focuses of IoT access technology [14]. With requirements of low complexity and power consumption, the transmission bandwidth of the NB-IoT physical channel is narrow, sometimes as little as one resource block (RB) [15]. In order to control the cost, a cheap and inaccurate crystal oscillator is always used, which leads to a large initial frequency offset for the system acquisition. M-part correlators and differential correlators can be used to mitigate the frequency offset effect on timing detection. In the work of Jun Zou and Hai Yu [16], a conjugated Zadoff–Chu (ZC) sequence based synchronization signal is fully studied in order to reduce timing drift caused by large frequency offset. In orthogonal frequency division multiplexing (OFDM) systems, timing is very important, so a lot of work has been done in this area, especially regarding the wideband system. Timing is also important to a narrowband system with a low sampling rate, because the low sampling rate means there will be a large sampling offset/delay, which can affect the timing performance.

As for timing synchronization in NB-IoT systems, a lot of research has been done. In [17], an effective method is proposed to reduce the complexity of NB-IoT cell search by splitting narrowband secondary synchronization signal (NSSS) generation formulas. In [18], frequency diversity (FD) reception for narrowband primary synchronization signals (NPSSs) and NSSSs is proposed to improve the physical-layer cell identity (PCID) detection probability. In [19], an NPSS detection method, whose timing metric is composed of symbol-wise autocorrelation and a dedicated normalization factor, is presented. In [20], hardware implementation of the maximum likelihood cross-correlation detection is presented to achieve low latency. However, until now, little research has been done relating to the improvement of timing accuracy. In this paper, we focus on the timing accuracy issue in the narrowband system with a low sampling rate and propose a fitting method to improve the timing accuracy with limited additional complexity.

In Section 2, we briefly describe the system model of the downlink synchronization procedure in the traditional wireless communication system. In Section 3, we propose a parabolic equation fitting algorithm to improve the timing accuracy. Section 4 presents simulation results for the verification of our design. Section 5 draws the conclusions.

## 2. System Model

At the transmitter, assuming the transmitted synchronization sequence is x[n], n=0,1,⋯,N−1, where *N* is the length of the synchronization sequence. We also assume that there is no data adjacent to the synchronization sequence, that is:

(1) x[n]=0, n≥N or n<0.

The transmitted continuous signal in the time domain can be written as:

(2) $$x_{a}(t)=\sum_{n=-\infty }^{\infty }x[n]\frac{\sin(\pi /T)(t-nT)}{\left(\pi /T)(t-nT\right)}$$

where $x[n]=x_{a}(nT)$ and T is the sample interval of the transmitted signal.

At the receiver, the received signal after sampling can be written as:

(3) $$y[n]=x_{a}(nT_{s}-n_{d}T_{s}-t_{d})+w[n]$$

where $T_{s}=T$ is the receiving sampling interval with the assumption that there is no frequency offset between the transmitter and the receiver, $n_{d}$ is the integer propagation delay, $t_{d}$ is the fractional propagation delay referred to as the sampling offset, and w[n] is the sampled noise.

In this paper, we focus on the sampling offset or the fractional propagation delay since the integer propagation delay does not affect the timing performance, which can be estimated by the synchronization algorithm, so we ignore $n_{d}$ in the following derivation. Therefore, Equation (3) can be simplified as:

(4) $$y[n]=x_{a}(nT_{s}-t_{d})+w[n]=\sum_{m=-\infty }^{+\infty }x[m]\frac{\sin[\pi (m-n-\tau )]}{\pi (m-n-\tau )}+w[n]=\sum_{m=-\infty }^{+\infty }x[m]Sa[\pi (m-n-\tau )]+w[n]$$

where $\tau =t_{d}/T_{s}$ is the normalized sampling offset and $Sa(x)≜\frac{\sin(x)}{x}$ is the sampling function. Without loss of generality, we can only consider the value of τ∈[0,1).

**Figure 1:**
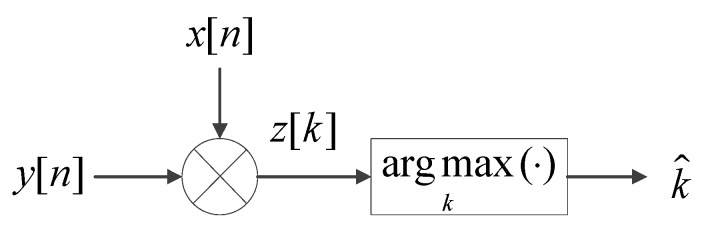
Illustrations of the synchronization signal detection with direct correlator.

In the traditional timing detection algorithm, a local sequence, which is a copy of the original synchronization sequence, is used to detect the timing of the received synchronization signal. The timing is determined by calculating the correlation between the received signal and the local sequence, as shown in Figure 1.

The output of the direct sliding correlator can be written as:

(5) $$z(k)=\frac{1}{N}\sum_{n=0}^{N-1}y[n+k]x^{*}[n].$$

Then a maximum likelihood estimate (MLE) is used to estimate the correct timing, which can be expressed as:

(6) $$\hat{k}=\underset{k}{\arg\max}\left|z(k)\right|.$$

Substituting Equation (4) into Equation (5) yields:

(7) $$\begin{array}{ll} z(k,\tau ) & =\frac{1}{N}\sum_{n=0}^{N-1}\left(\sum_{m=0}^{N-1}x[m]Sa[\pi (m-n-k-\tau )]\right)x^{*}[n]+\frac{1}{N}\sum_{n=0}^{N-1}w[n+k]x^{*}[n]\\ & =\frac{1}{N}\sum_{n=0}^{N-1}\sum_{m=0}^{N-1}x[m]x^{*}[n]Sa[\pi (m-n-k-\tau )]+v_{1}\left(k,\tau \right) \end{array}$$

where

(8) $$v_{1}\left(k,\tau \right)=\frac{1}{N}\sum_{n=0}^{N-1}w[n+k]x^{*}[n]$$

is the noise related term that obeys the normal distribution since the w[m] is the white Gaussian noise with zero mean. Then we focus on the first term in Equation (7) without considering the noise effect, which can be rewritten as:

(9) $$z(k,\tau )=\frac{1}{N}\sum_{n=0}^{N-1}\sum_{m=0}^{N-1}x[m]x^{*}[n]Sa[\pi (m-n-k-\tau )].$$

For a given k and τ, Equation (9) can be considered as a sum of different sampling functions decided by m and n. When m−n is determined, Sa[π(m−n−k−τ)] is a deterministic value, so we can rearrange the term order in Equation (9) as:

(10) $$\begin{array}{ll} z(k,\tau ) & =\frac{1}{N}\sum_{p=-N+1}^{N-1}\left(\sum_{n=0}^{N-1}x[n+p]x^{*}[n]Sa[\pi (p-k-\tau )]\right)\\ & =\sum_{p=-N+1}^{N-1}Sa[\pi (p-k-\tau )]\left(\frac{1}{N}\sum_{n=0}^{N-1}x[n+p]x^{*}[n]\right)\\ & =\sum_{p=-N+1}^{N-1}Sa[\pi (p-k-\tau )]r_{1}(p) \end{array}$$

where p=m−n and $r_{1}(p)=\frac{1}{N}\sum_{n=0}^{N-1}x[n+p]x^{*}[n]$, which is the normalized output of the direct sliding correlator.

For the cyclic corrector, such as the preamble detection in the Long Term Evolution (LTE) uplink [1], the output of the cyclic correlator can be written as:

(11) $$z(k)=\frac{1}{N}\sum_{n=0}^{N-1}y{\langle n+k\rangle }_{N}x^{*}[n]=\frac{1}{N}\sum_{n=0}^{N-1}y[n]x^{*}{\langle n-k\rangle }_{N}$$

where $x{\langle n-k\rangle }_{N}$ represents a cyclic shift sequence.

Substituting Equation (4) into Equation (11) yields:

(12) $$\begin{array}{ll} z(k,\tau ) & =\frac{1}{N}\sum_{n=0}^{N-1}\left(\sum_{m=0}^{N-1}x[m]Sa[\pi (m-n-\tau )]\right)x^{*}{\langle n-k\rangle }_{N}+\frac{1}{N}\sum_{n=0}^{N-1}w[n]x^{*}{\langle n-k\rangle }_{N}\\ & =\frac{1}{N}\sum_{n=0}^{N-1}\sum_{m=0}^{N-1}x[m]x^{*}{\langle n-k\rangle }_{N}Sa[\pi (m-n-\tau )]+v_{2}(k,\tau ) \end{array}$$

where $v_{2}(k,\tau )=\frac{1}{N}\sum_{n=0}^{N-1}w[n]x^{*}{\langle n-k\rangle }_{N}$ is the noise related term. Without considering the noise term, Equation (12) can be rewritten as:

(13) $$z(k,\tau )=\frac{1}{N}\sum_{n=0}^{N-1}\sum_{m=0}^{N-1}x[m]x^{*}{\langle n-k\rangle }_{N}Sa[\pi (m-n-\tau )].$$

In addition, Equation (13) can be rewritten as:

(14) $$\begin{array}{ll} z(k,\tau ) & =\frac{1}{N}\sum_{p=0}^{N-1}\left(\sum_{n=0}^{N-1}x{\langle n+p\rangle }_{N}x^{*}[n]Sa[\pi (p-k-\tau )]\right)\\ & =\sum_{p=0}^{N-1}Sa[\pi (p-k-\tau )]\left(\frac{1}{N}\sum_{n=0}^{N-1}x{\langle n+p\rangle }_{N}x^{*}[n]\right)\\ & =\sum_{p=0}^{N-1}Sa[\pi (p-k-\tau )]r_{2}(p) \end{array}$$

where $r_{2}(p)=\frac{1}{N}\sum_{n=0}^{N-1}x{\langle n+p\rangle }_{N}\cdot x^{*}[n]$ is the normalized output of the cyclic correlator.

By comparing Equation (10) and Equation (14), we can see that the effect of sampling offset is to make the correlator output be the summation of correlations weighted by the sampling function.

When the correlator output is ideal, that is:

(15) $$r_{i}[p]=\left\{ \begin{array}{ll} 1 & \;p=0\;\\ 0 & \;\mathrm{others} \end{array}\right.,$$

substituting Equation (15) into Equations (10) and (14) yields

(16) z(k,τ)=Sa[π(k+τ)].

According to Equation (16), we can see that the output of the correlator is a perfect sampling function under ideal conditions. When chosing a designed sequence, the cyclic correlator output can satisfy the ideal condition, such as the ZC sequence. However, the sliding correaltor output in practical situations cannot avoid sidelobes when p≠0, but they are usually much smaller compared with the peak.

The ZC sequence is chosen in the following analysis because of its perfect autocorrelation property. When the sequence length is large, sidelobes in the sliding correlation are also quite small. The ZC sequence can be expressed as [21]:

(17) $$x[n]=e^{-j\frac{\pi \mu n(n+1)}{N}},\;n=0,1,2,\cdots N-1$$

where N is the length of the ZC sequence and μ=1,2,⋯,N−1 is the root of the ZC sequence.

**Figure 2:**
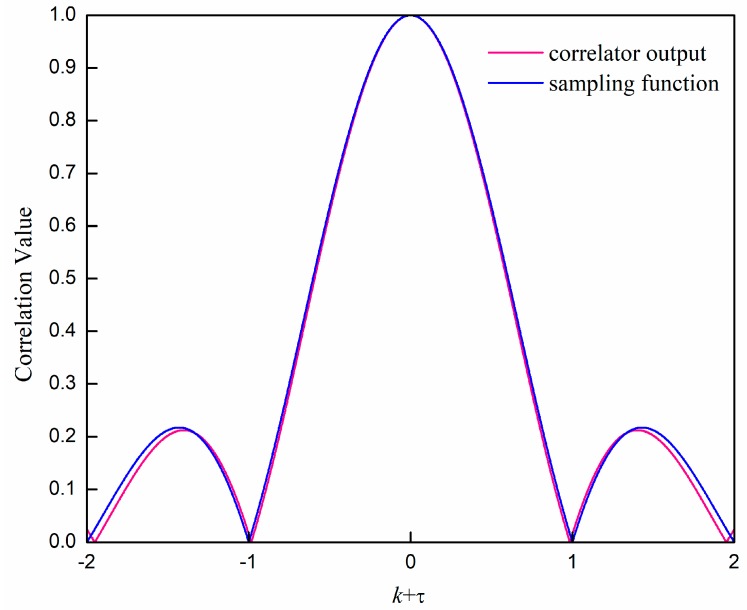
The output of the direct sliding correlator with the Zadoff–Chu (ZC) sequence, μ=25 and N=64.

Figure 2 shows the output of the direct sliding correlator with a ZC sequence according to Equation (10). The length of the ZC sequence is 64, and the root index is 25. We can also add the sampling function as a reference. We can see that the gap between these two curves is rather small, especially the central part from −1 to 1. This means that the influence of correaltor output sidelobes is small in this case, which provides a theoretical basis for the optimization method that we will propose in the following.

## 3. Parabolic Equation Fitting Timing

**Figure 3:**
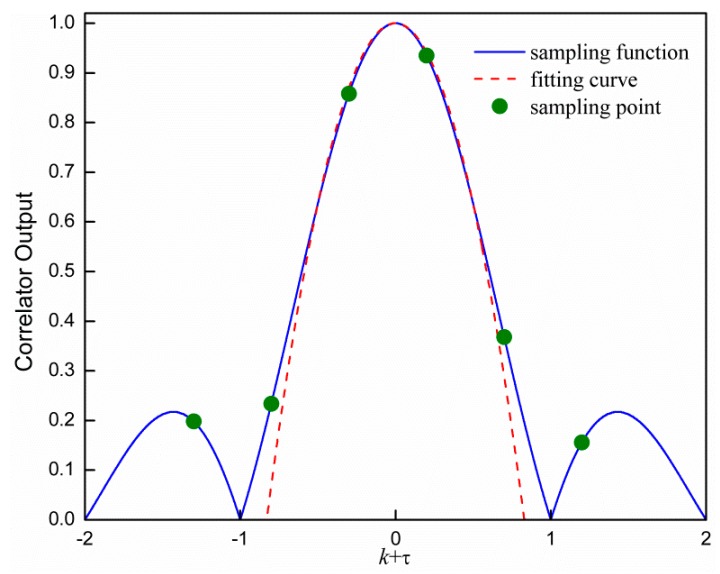
Illustrations of the direct sliding correlator output with a sampling rate two times that of the Nyquist sampling rate.

From the previous analysis, we know that the output of the direct correlator with the ZC sequence is approximate to the sampling function. Due to the uncertainty of signal arrival, the actual sampling point cannot always be the peak of the sampling function, which also leads to the decrease of the largest correlator output, as shown in Figure 3. In Figure 3, the ZC sequence is used with the same parameters as Figure 2, and the sampling rate is two times that of the Nyquist sampling rate. We can also add the sampling function as a reference.

It is of great importance to find the true peak, making use of the points influenced by the sampling offset. The range of sampling offset or quantization error is $(-\frac{1}{2f_{s}},\frac{1}{2f_{s}})$, where $f_{s}=1/T_{s}$ is the sampling rate. Considering that the sampling function is hard to fit since its derivative is too complex, parabola is a better choice to fit the correlator output curve when what we are looking at are just the values near the peak.

The correlator output curve can be fitted by parabola with a general form:

(18) $$z-v=ax^{2}+bx+c$$

where z is the correlator output, v is the noise, and x is the time.

According to the property of parabola, the real peak is:

(19) $$x_{\mathrm{peak}}=-\frac{b}{2a}.$$

Assuming there are N points that we know, i.e., $\left(x_{1},z_{1}\right),\left(x_{2},z_{2}\right)\cdot \cdot \cdot \left(x_{N},z_{N}\right)$, where $x_{i}$ is the time and $z_{i}$ is the correlator output, Equation (18) can be rewritten in matrix form as:

(20) Z=AS+V

where $Z=\left[\begin{array}{l} z_{1}\\ z_{2}\\ \vdots \\ z_{N} \end{array}\right]$, $A=\left[\begin{array}{lll} x_{1}{}^{2} & x_{1} & 1\\ x_{2}{}^{2} & x_{2} & 1\\ \vdots & \vdots & \vdots \\ x_{N}{}^{2} & x_{N} & 1 \end{array}\right]$, $S=\left[\begin{array}{l} a\\ b\\ c \end{array}\right]$, $V=\left[\begin{array}{l} v_{1}\\ v_{2}\\ \vdots \\ v_{N} \end{array}\right]$, Z is the correlator output determinant, A is the coefficient matrix of time, S is the solution determinant, and V is the noise determinant.

In the practical system, v is usually hard to estimate at the downlink synchronization procedure, so minimum mean square error (MMSE) is not considered in this paper. Therefore, the least-square (LS) estimation method is a better choice. The error function is given as:

(21) $$J={\left(Z-\hat{Z}\right)}^{H}(Z-\hat{Z})={\left(Z-A\hat{S}\right)}^{H}(Z-A\hat{S}).$$

The purpose of LS is to minimize J. So we take the derivative of J and set it to zero, that is:

(22) $$\frac{\partial \left\{{\left(Z-A\hat{S}\right)}^{H}(Z-A\hat{S})\right\}}{\partial \hat{S}}=0.$$

Then we can get:

(23) $$\hat{S}={\left(A^{H}A\right)}^{-1}A^{H}Z.$$

Therefore, when the inverse matrix of A exists, Equation (23) can be rewritten as:

(24) $$\hat{S}=A^{-1}Z.$$

However, the number of rows and columns of a matrix cannot be guaranteed to be the same, e.g., the number of points, N, is larger than three, which means the inverse matrix does not exist, so we need to form a square matrix, making use of A. Multiplying both sides by the transpose of matrix A, Equation (20) can be rewritten as:

(25) $$A^{T}AS=A^{T}Z+A^{T}V$$

where $A^{T}$ represents the transpose of matrix A. $A^{T}A$ is a N×N square matrix, but what is important is that even a square matrix is not determined to own inverse matrix; the algorithm above is not stable but is applicable to most conditions.

What we need is a method that is applicable in all situations. Based on the LS method, generalized inverse matrix theory is an effective method to acquire the least-square solution. The generalized inverse matrix, $A^{+}$, can be easily calculated by singular value decomposition (SVD). N×3 matrix A can be rewritten by SVD as:

(26) $$A=U{\left(\begin{array}{ll} \sum & 0\\ 0 & 0 \end{array}\right)}_{N\times 3}V^{H}$$

where the unitary matrix, V, $\sum ≜diag(\sigma_{1},\sigma_{2}\cdots \sigma_{r})$, $\sigma_{1},\sigma_{2}\cdots \sigma_{r}$, is the singular value, and the generalized inverse matrix of A is:

(27) $$A^{+}=V{\left(\begin{array}{ll} \sum^{-1} & 0\\ 0 & 0 \end{array}\right)}_{3\times N}U^{H}.$$

For a contradictory equation, A⋅S=Y, the application of the LS method is to find a solution to minimize ${\|A\cdot s-Y\|}_{2}$, where s is a single value of its determinant. The least-norm solution of the contradictory equation is:

(28) $$S=A^{+}Z.$$

The solution in Equation (28) can be adopted for any number of points. There is no doubt that the more points used in the optimization, the closer we get to what we want in theory, but this will increase the computing complexity. Three points can decide a parabola, so the sampling rate is at least two times that of the Nyquist sampling rate, which ensures that three points with the maximum correlator output lie in the central parabola part, i.e., k+τ∈[−0.5,0.5]. When only three points can be used, we can calculate the estimated timing with a closed form.

Assuming the three points are the points with the maximum correlator output $\left(t_{2},\;Z_{2}\right)$ and its two adjacent points $\left(t_{1},\;Z_{1}\right)$ and $\left(t_{3},\;Z_{3}\right)$, $Z_{i}$ is the correlator output corresponding to $t_{i}$, which satisfies:

(29) $$t_{3}-t_{2}=t_{2}-t_{1}=\frac{1}{f_{s}}.$$

The coefficient of the parabola can be calculated by:

(30) $$\begin{array}{l} a=\frac{\left(Z_{1}-Z_{2})(t_{2}-t_{3})-(Z_{2}-Z_{3})(t_{1}-t_{2}\right)}{\left(t_{1}^{2}-t_{2}^{2})(t_{2}-t_{3})-(t_{2}^{2}-t_{3}^{2})(t_{1}-t_{2}\right)}=\frac{\left(Z_{1}+Z_{3}-2Z_{2}\right)}{2}f_{s}^{2}\\ b=\frac{\left(Z_{1}-Z_{2}\right)(t_{2}^{2}-t_{3}^{2})-\left(Z_{2}-Z_{3}\right)(t_{1}^{2}-t_{2}^{2})}{\left(t_{2}^{2}-t_{3}^{2})(t_{1}-t_{2})-(t_{1}^{2}-t_{2}^{2})(t_{2}-t_{3}\right)}=\frac{\left(Z_{1}-Z_{2}\right)(t_{2}^{2}-t_{3}^{2})-\left(Z_{2}-Z_{3}\right)(t_{1}^{2}-t_{2}^{2})}{2}f_{s}^{3} \end{array}$$

Then the estimated timing point is:

(31) $$\hat{t}=-\frac{b}{2a}=\frac{\left(Z_{1}-Z_{2}\right)(t_{2}^{2}-t_{3}^{2})-\left(Z_{2}-Z_{3}\right)(t_{1}^{2}-t_{2}^{2})}{2(Z_{2}-Z_{3})(t_{1}-t_{2})-2(Z_{1}-Z_{2})(t_{2}-t_{3})}=\frac{\left(Z_{1}-Z_{2}\right)(t_{2}^{2}-t_{3}^{2})-\left(Z_{2}-Z_{3}\right)(t_{1}^{2}-t_{2}^{2})}{4Z_{2}-2Z_{1}-2Z_{3}}f_{s}.$$

There are just ten additional multiplication operations in Equation (31) compared with the traditional timing method. Moreover, in the traditional LTE downlink synchronization procedure, the sampling rate is usually set to two times that of the Nyquist sampling rate. Therefore, the additional complexity of our proposed method is quite small.

## 4. Simulation Results

In this section, the simulation results are given to verify the performance of our proposed timing optimization method based on parabolic equation fitting. The system parameters are set as follows: The synchronization signal was generated in the frequency domain in the OFDM system, the subcarrier spacing was $\Delta f_{s}=3$ kHz, the length of the ZC sequence was N=64, the Nyquist sampling rate was 192 kHz, the root of the ZC sequence was μ=25, and the synchronization signal period was 10 ms. The additive white Gaussian noise (AWGN) channel was used in the simulations.

**Figure 4:**
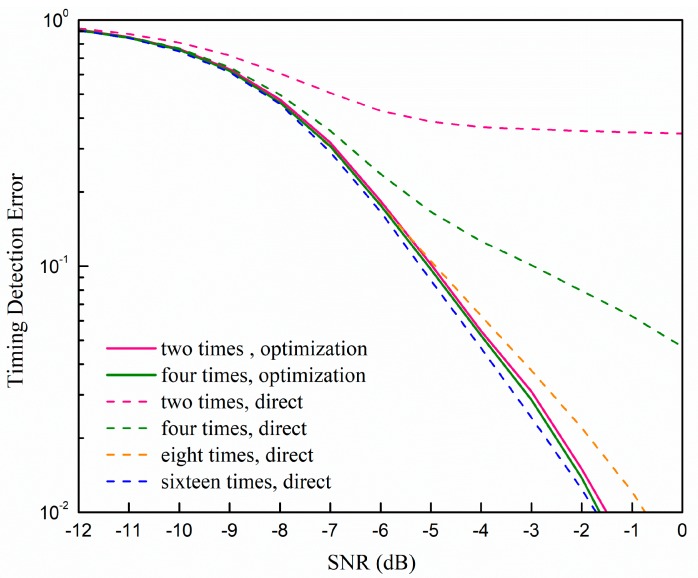
Comparisons of the timing detection error rate with different sampling rates and methods at different signal-to-noise (SNR).

Figure 4 shows the comparison between the timing detection error rate of the direct sliding correlator and the parabolic equation fitting optimization with different signal-to-noise ratios (SNRs). The sampling rate of the parabolic equation fitting method was set to two times and four times that of the Nyquist sampling rate. The higher sampling rate without the parabolic equation fitting is also added as a reference. We regard it as a detection error when the timing error is larger than 0.15 μs. We can see that our proposed method can work much better than the direct correlator with two times the rate of the Nyquist sampling rate, which is close to the performance of the direct correlator with sixteen times the rate of the Nyquist sampling rate. Moreover, the performance of the parabolic equation fitting optimization with four times the rate of the Nyquist sampling rate is nearly the same as the performance with two times the rate of the Nyquist sampling rate. The points according to Equation (29) are used in the scenario that is four times the rate of the Nyquist sampling rate scenario; the reason will be discussed in detail in the following.

**Figure 5:**
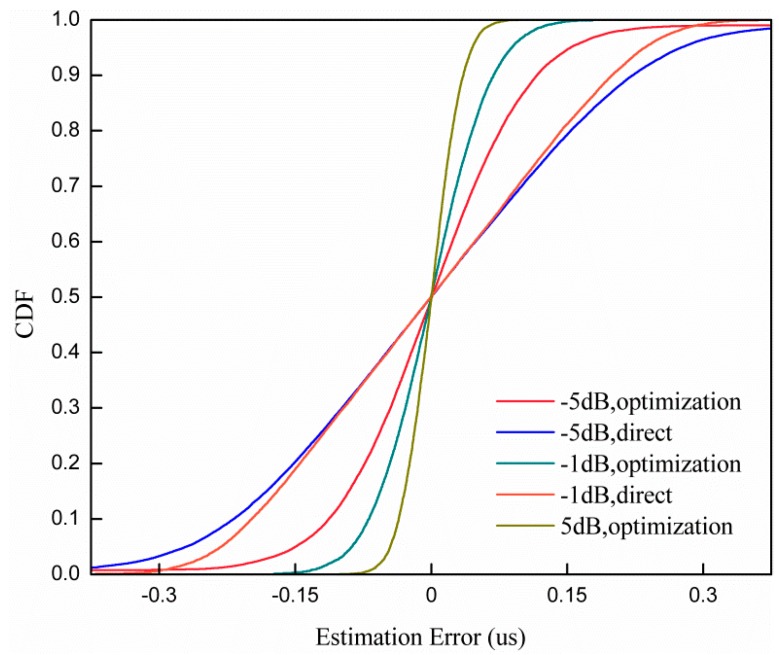
Comparisons between the cumulative distribution function (CDF) of the timing detection error rate of the parabolic equation fitting method and the direct correlator.

Figure 5 shows the comparisons between the cumulative distribution function (CDF) of the timing detection error rate with different synchronization methods and SNRs. We can see that the slope of our proposed method is much steeper than the slope of the direct correlator at the same SNR. As the SNR increases, the gap between the proposed method and the direct correlator increases since the effect of noise on parabolic equation fitting becomes small as the SNR increases.

**Figure 6:**
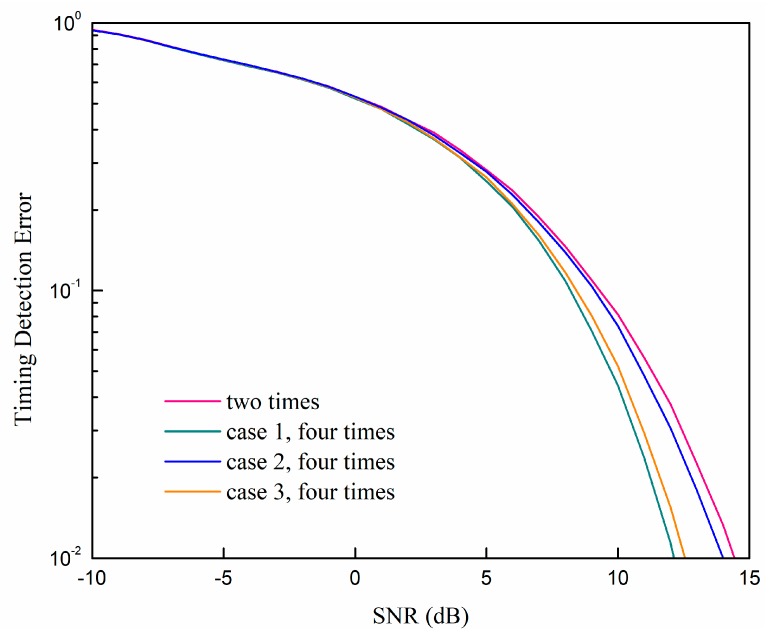
Timing detection error rate of parabolic equation fitting with four times the rate of the Nyquist sampling rate using different point combinations.

Figure 6 shows the timing detection error rate of the proposed method with four times the rate of the Nyquist sampling rate with the different point selection method. For showing the gap between different selection methods more clearly, we have determined that a detection error has occurred when the timing error is larger than 0.02 μs. When the sampling rate is four times that of the Nyquist sampling rate, there are at least eight points in the main lobe of the sampling function. We focus on the five points around the maximum output of the direct correlator. We assume that the five points are $\left(t_{i},\;Z_{i}\right),i=1,2,3,4,5$, and the point with the maximum output of the direct correlator is $\left(t_{3},\;Z_{3}\right)$, both of which satisfy:

(32) $$t_{5}-t_{4}=t_{4}-t_{3}=t_{3}-t_{2}=t_{2}-t_{1}=\frac{1}{f_{s}}$$

**Figure 7:**
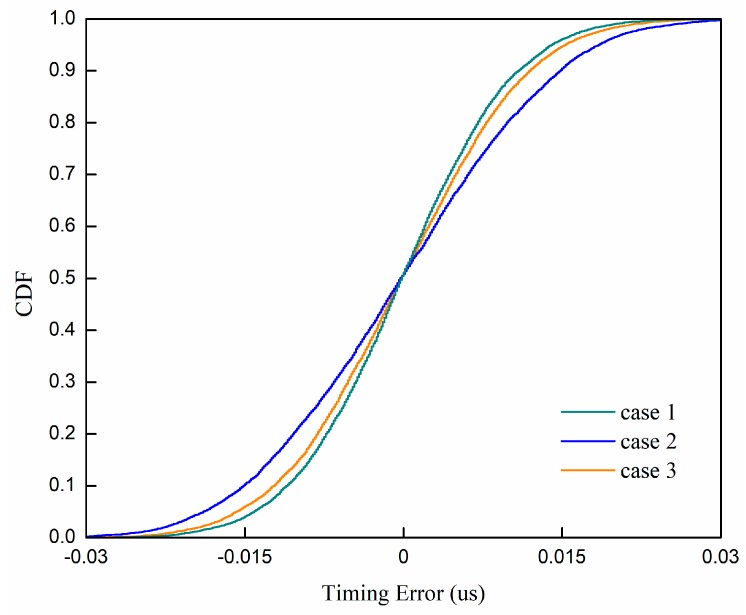
CDF comparisons of the timing detection error rate of the parabolic equation fitting with four times the rate of the Nyquist sampling rate.

**Figure 8:**
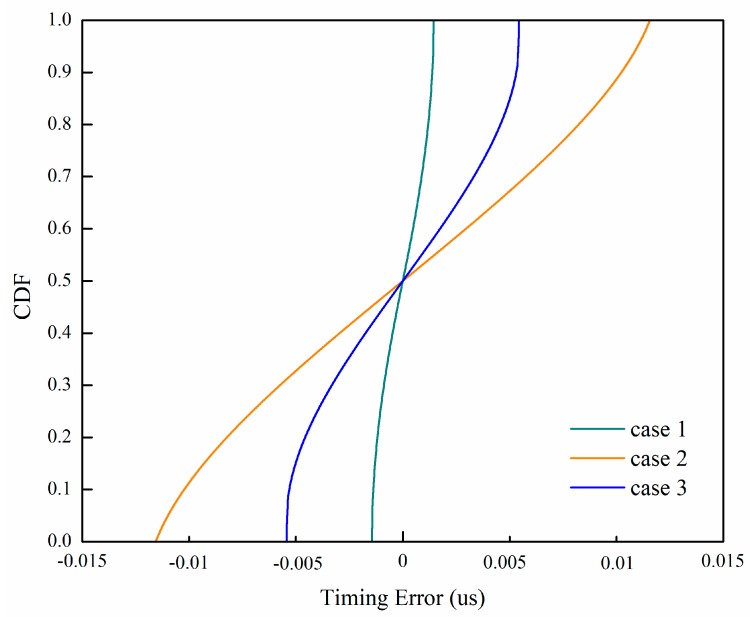
CDF comparisons of the timing detection error rate of the parabolic equation fitting with four times the rate of the Nyquist sampling rate without noise.

In Figure 6, we have simulated three situations: (1) The point with the maximum correlator output $\left(t_{3},\;Z_{3}\right)$ and its neighbors $\left(t_{2},\;Z_{2}\right)$ and $\left(t_{4},\;Z_{4}\right)$; (2) the point with the maximum correlator output $\left(t_{3},\;Z_{3}\right)$ and $\left(t_{1},\;Z_{1}\right)$$\left(t_{5},\;Z_{5}\right)$; (3) all five points. From Figure 6, we can see that case 1 is the best. We also add the proposed method with two times the rate of the Nyquist sampling rate as a reference, which is similar to case 2. Figure 7 shows the CDF comparisons of the timing detection error rate with different selection methods when the SNR is 10 dB. Figure 8 shows the CDF comparisons of the timing detection error rate with different selection methods when there is no noise. From Figure 8, we can see that the performance of case 1 is the best, which means it is the closet to the sampling function. It is consistent with the performance in Figure 6.

**Figure 9:**
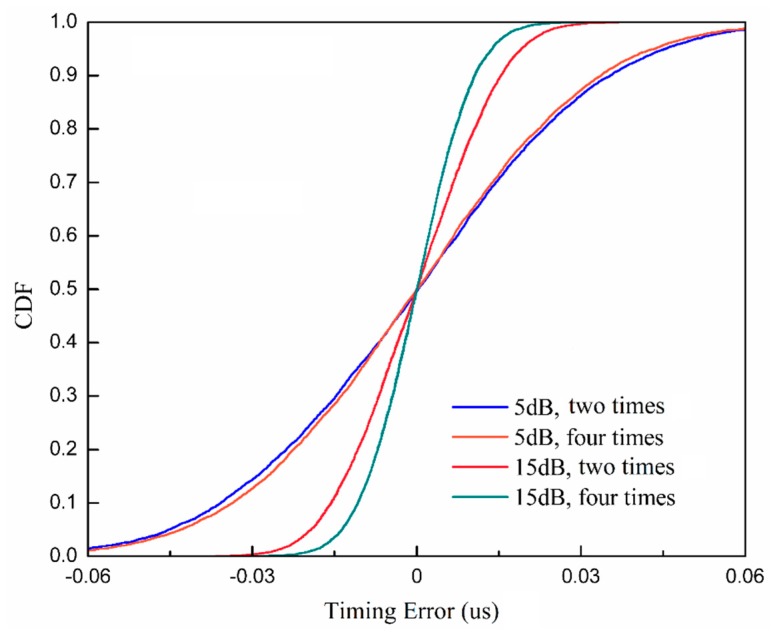
CDF comparisons of the timing detection error rate of the parabolic equation fitting with two times the rate and four times the rate of the Nyquist sampling rate.

Figure 9 shows the CDF comparisons of detection error rate with different sampling rates and SNRs. We can see that when the SNR is 5 dB, the gap between the two times rate and the four times rate of the Nyquist sampling rate is very small. As SNR increases to 10 dB, the improvement is obvious. It means that two times the rate of the Nyquist sampling rate is enough in the low SNR region. When the SNR is high, we can improve the performance by increasing the sampling rate with the increase of computation complexity.

**Figure 10:**
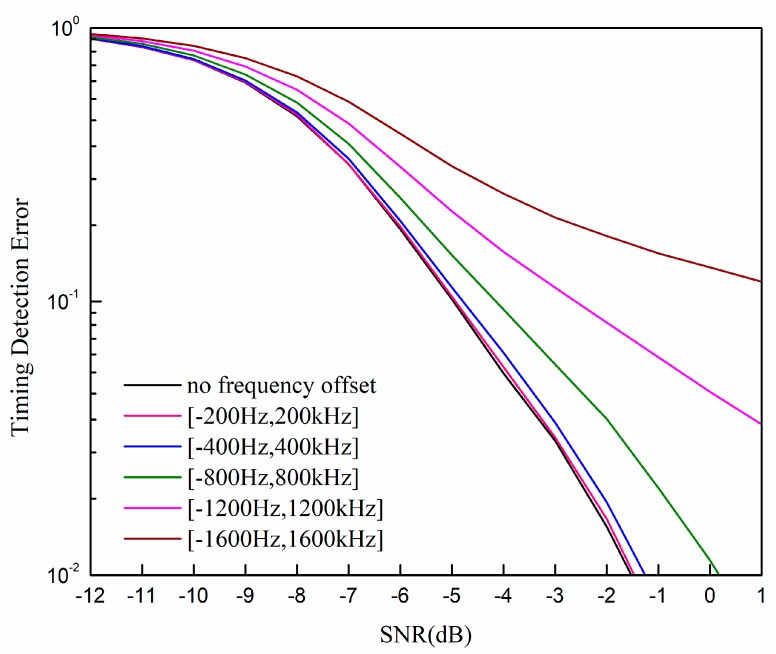
Timing detection error rate of our proposed method with different frequency offsets.

Figure 10 shows the performance of our proposed method with different frequency offsets. The sampling rate is two times that of the Nyquist sampling rate, and the frequency offset is randomly and uniformly added. We can see that when the frequency is small, e.g., smaller than 400 Hz, the performance degeneration is small. When the frequency offset is up to 800 Hz, the performance degeneration becomes large. The reason for this is that our proposed method is based on the outputs of the direct correlator, whose performance is affected by the frequency offset. In general, our proposed method is not sensitive to the frequency offset when the frequency offset is in the tolerance range of the direct correlator.

## 5. Conclusions

In this paper, we investigated a method to improve the timing precision in narrowband systems. The sampling offset was small in the wideband system due to the high sampling rate, but in the narrowband system it was a nonignorable factor, especially when the sampling rate was low. After considering the sampling offset, the output of the direct correlator, which is widely used to accomplish downlink synchronization, was a summation of traditional correlator outputs weighted by sampling function. So, we proposed a parabolic equation fitting method to improve the timing precision with little additional complexity. The simulation results showed that the performance of our proposed parabolic equation fitting method with two times the rate of the Nyquist sampling rate was close to the traditional direct correlator method with sixteen times the rate of the Nyquist sampling rate. In addition, we found that two times the rate of the Nyquist sampling rate is enough when the SNR is low. Therefore, we could increase the timing precision by increasing ten additional multiplication operations based on the output of the traditional direct correlator.
